# Supplementary material for: Combined person classification with airborne optical sectioning
Source: Sci Rep. 2022 Mar 9;12:3804. doi: 10.1038/s41598-022-07733-z (PMC8907346; doi:10.1038/s41598-022-07733-z)
Supplement: Supplementary file 1 — Supplementary Information. [file 41598_2022_7733_MOESM1_ESM.pdf]

Supplementary Material:

Combined Person Classification with Airborne Optical Sectioning

Indrajit Kurmi[1], David C. Schedl[1], & Oliver Bimber[1,*]

[1]*Johannes Kepler University, Institute of Computer Graphics, Linz, 4040, Austria*

[*] oliver.bimber@jku.at

S1 Derivation of Integrated Occlusion Density

Here we present the derivation of integrated occlusion density $\widetilde{D_\alpha}$, equation (7), for a ray passing through an occlusion volume at an angle α based on the statistical model described in[1]. Integrated occlusion density $\widetilde{D}$ for a ray of length l passing orthogonally ($\alpha = 0°$) through the occlusion volume of density D, height l, and filled with occluders of uniform distribution and size o is[1]:

$$\widetilde{D} = 1 - (1 - D)^{l/o}. \tag{S1}$$

The length of a ray passing at an oblique angle α (α > *0*°) through the same occlusion volume is $l/(cos(\alpha))$, and substituting this in equation (S1) yields:

$$\widetilde{D_\alpha} = 1 - (1 - D)^{l/cos(\alpha)o}. \tag{S2}$$

Applying the logarithm after simplifying both equations (S1) and (S2) respectively leads to:

$$log\,(1 - \widetilde{D}) = \frac{l}{o} log\,(1 - D) \tag{S3}$$

and

$$log\,(1 - \widetilde{D_\alpha}) = \frac{l}{cos(\alpha)o} log\,(1 - D). \tag{S4}$$

Substituting equation (S3) in (S4) yields:

$$log\,(1 - \widetilde{D_\alpha}) = \frac{1}{cos(\alpha)} log\,(1 - \widetilde{D}). \tag{S5}$$

Transforming the logarithmic equation in equation (S5) to its equivalent exponential form and simplifying yields (7).

S2 Enlarged View of Figure 4 and Figure 5

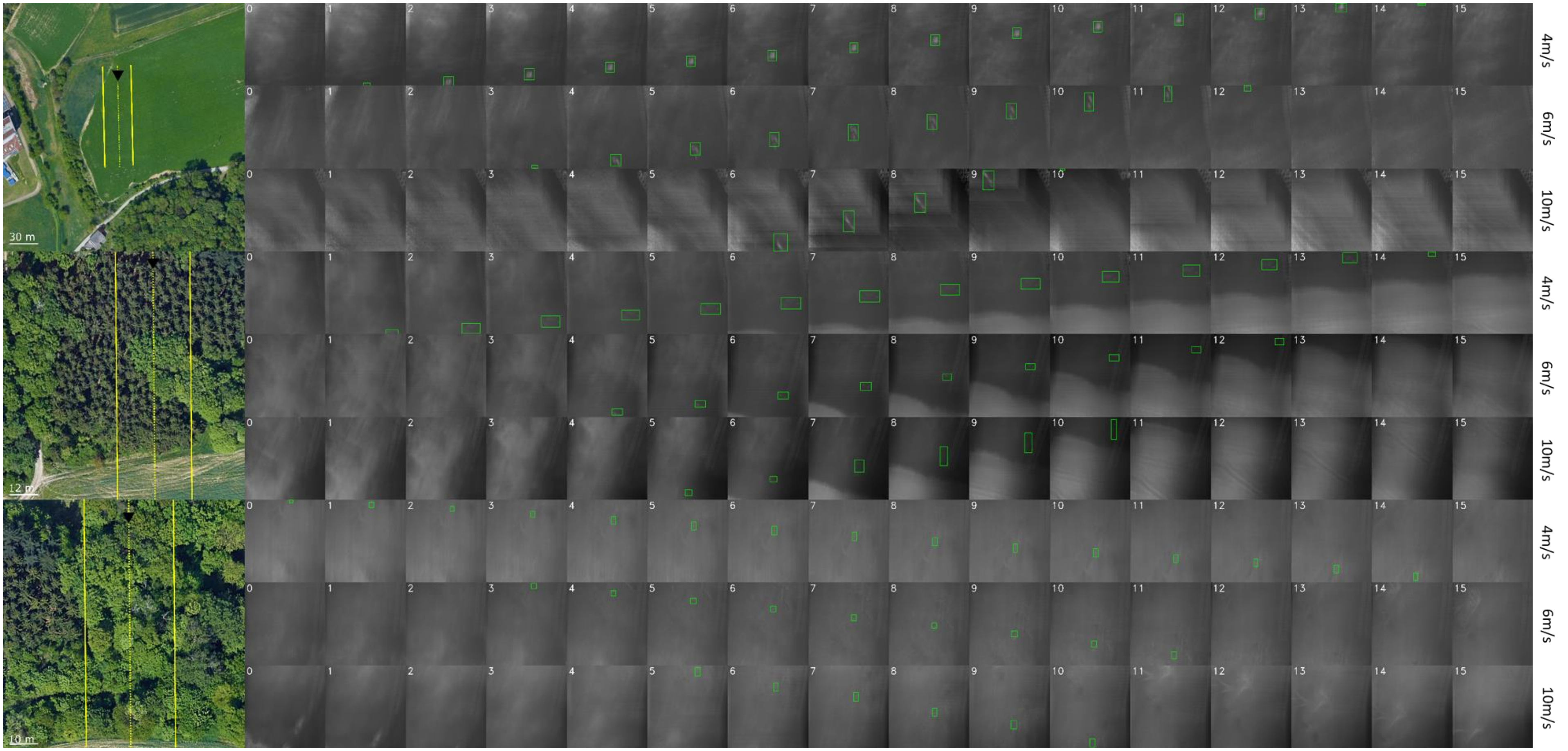

Test sites, flight directions, and coverages (left) at constant flying speeds of $v_f = 4$, 6, and 10 m/s over unoccluded (open field) terrain (top rows), over conifer forest (middle rows), and over broadleaf forest (bottom rows). Resulting integral images (right) reveal the appearance of the same person (bounding boxes indicate the manually labeled ground-truth appearances) of $o_\int = 13.8$ for 4 m/s, 9.2 for 6 m/s, and 5.5 for 10 m/s. Note that the shape of the ground-truth bounding boxes varies slightly due to miss-registration in the integral images. The GPS coordinates of the test sites are: 48°20'08.4"N, 14°19'34.6"E (open field), 48°19'58.1"N 14°19'48.1"E (conifer forest), 48°19'59.8"N 14°19'52.2"E (broadleaf forest).

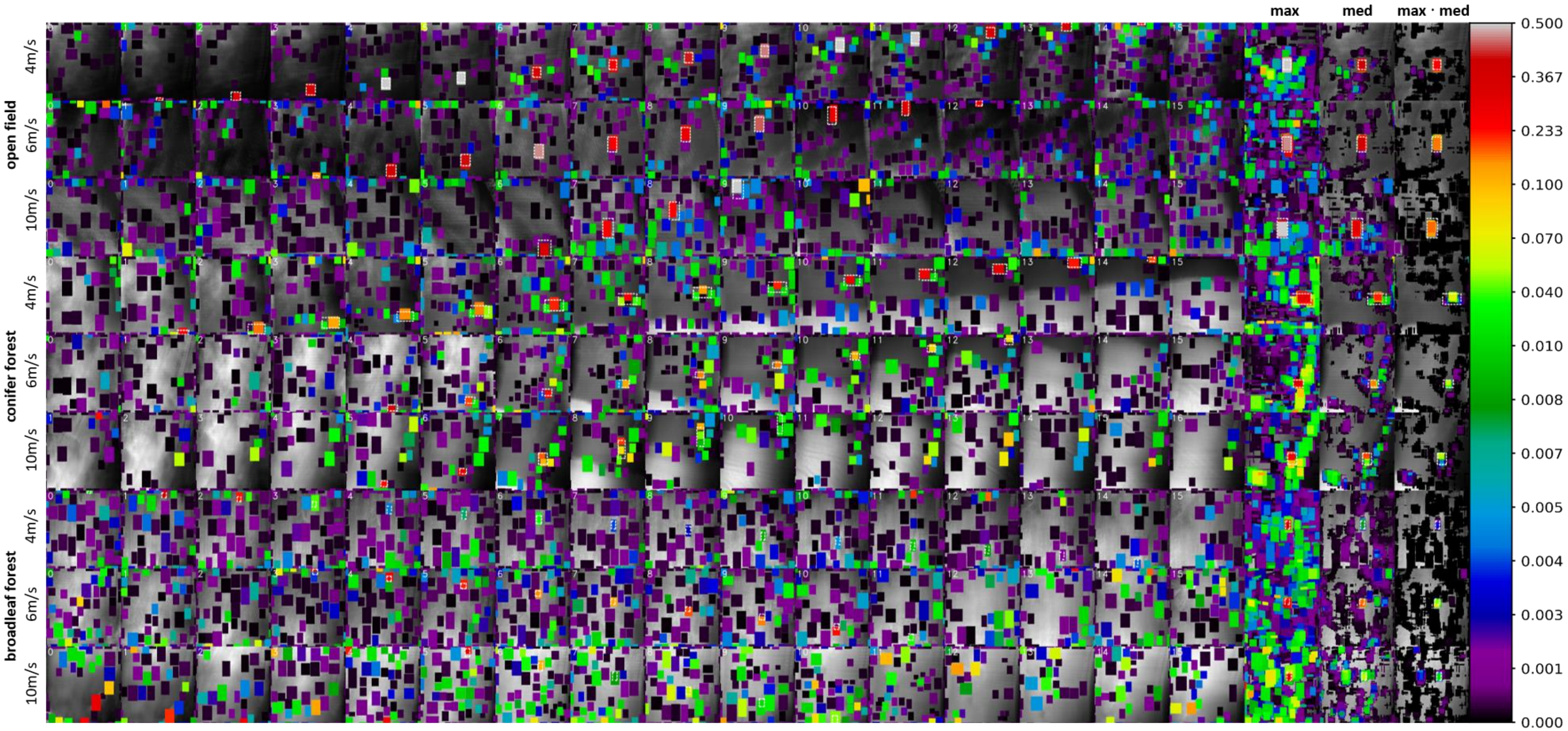

Probability maps of single integral images (first 16 columns) and combined classification results (last three columns: maximum, median, and maximum·median) for all test flights. Detections are indicated with AABBs, and confidence scores are color coded (see the logarithmic color bar on the right).

References

1. I Kurmi, D C Schedl, and O Bimber. “A statistical view on synthetic aperture imaging for occlusion removal”. *IEEE Sensors J* **19**, 9374–9383 (2019).
